# (*R*,*R*)-4,4′-Dibromo-2,2′-[cyclo­hexane-1,2-diylbis(nitrilo­methyl­idyne)]diphenol

**DOI:** 10.1107/S1600536809039671

**Published:** 2009-10-03

**Authors:** Jianhong Yi, Shuangqi Hu

**Affiliations:** a School of Material Science and Engineering, North University of China, Taiyuan 030051, People’s Republic of China; b School of Chemical Engineering and Environment, North University of China, Taiyuan 030051, People’s Republic of China

## Abstract

The mol­ecule of the title compound, C_20_H_20_Br_2_N_2_O_2_, lies on a twofold axis. It contains two stereogenic C atoms with *R* chirality and thus it is the enatiomerically pure *R*,*R*-diastereomer. There is an intra­molecular O—H⋯N hydrogen bond.

## Related literature

For the structure of 1,2-cyclo­hexa­nediamine, see: Yang *et al.*, (2004 ▶, 2007 ▶). For background to the use of chiral Salen compounds containing the 1,2-cyclo­hexa­nediamine motif in asymmetric catalytic synthesis, see: Canail & Sherrington (1999 ▶); Jacobsen (2000 ▶). 
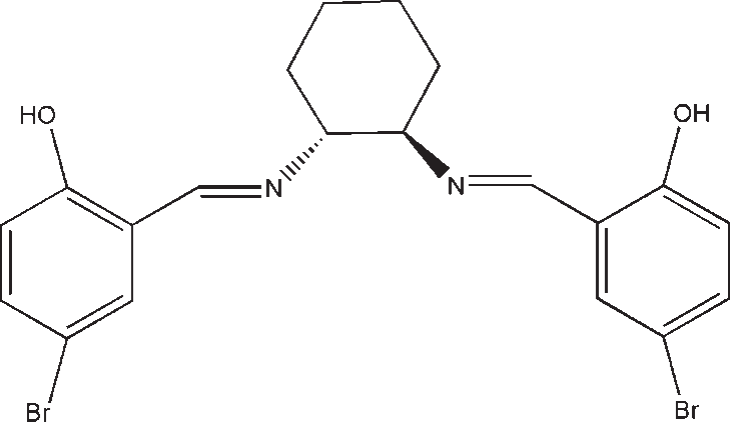


## Experimental

### 

#### Crystal data

C_20_H_20_Br_2_N_2_O_2_
*M* *_r_* = 480.20
Orthorhombic, 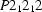

*a* = 5.9323 (16) Å
*b* = 19.079 (5) Å
*c* = 9.009 (2) Å
*V* = 1019.7 (4) Å^3^
*Z* = 2
Mo *K*α radiation
μ = 3.99 mm^−1^
*T* = 298 K
0.28 × 0.21 × 0.15 mm

#### Data collection

Bruker APEXII area-detector diffractometer
Absorption correction: multi-scan (*SADABS*; Bruker, 2005 ▶) *T*_min_ = 0.401, *T*_max_ = 0.586
5912 measured reflections
1727 independent reflections
1449 reflections with *I* > 2σ(*I*)
*R*_int_ = 0.024

#### Refinement

*R*[*F* ^2^ > 2σ(*F* ^2^)] = 0.032
*wR*(*F* ^2^) = 0.089
*S* = 1.06
1727 reflections
119 parameters
H-atom parameters constrained
Δρ_max_ = 0.34 e Å^−3^
Δρ_min_ = −0.39 e Å^−3^
Absolute structure: Flack (1983 ▶), 681 Friedel pairs
Flack parameter: 0.018 (18)

### 

Data collection: *APEX2* (Bruker, 2005 ▶); cell refinement: *SAINT* (Bruker, 2005 ▶); data reduction: *SAINT*; program(s) used to solve structure: *SHELXS97* (Sheldrick, 2008 ▶); program(s) used to refine structure: *SHELXL97* (Sheldrick, 2008 ▶); molecular graphics: *ORTEPIII* (Burnett & Johnson, 1996 ▶) and *ORTEP-3 for Windows* (Farrugia, 1997 ▶); software used to prepare material for publication: *SHELXL97*.

## Supplementary Material

Crystal structure: contains datablocks I, global. DOI: 10.1107/S1600536809039671/dn2493sup1.cif

Structure factors: contains datablocks I. DOI: 10.1107/S1600536809039671/dn2493Isup2.hkl

Additional supplementary materials:  crystallographic information; 3D view; checkCIF report

## Figures and Tables

**Table 1:** Hydrogen-bond geometry (Å, °)

*D*—H⋯*A*	*D*—H	H⋯*A*	*D*⋯*A*	*D*—H⋯*A*
O1—H1⋯N1	0.82	1.91	2.611 (4)	143

**Fig. 1.**
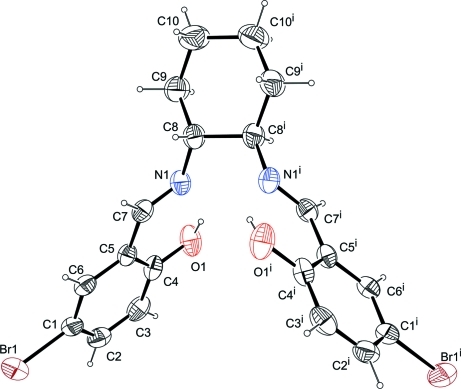
Molecular view of (I) with the atom-labeling scheme. Ellipsoids are drawn at the the 30% probability level. H atoms are represented as small spheres of arbitrary radii. [Symmetric code: (i): -x+1, -y+2, z]

## supplementary crystallographic information

### Comment

Chiral Salen compounds containing 1,2-cyclohexanediamine motif are widely used in the asymmetric catalytic synthesis (Canail & Sherrington, 1999; Jacobsen *et al.*, 2000). Until now, only few single-crystal structures of chiral Salen compounds were reported. Some interesting compounds with 1,2-cyclohexanediamine have however been reported (Yang *et al.*, 2004; 2007). In an attempt to form a Cd(II) complex with the (R,R)-4,4'-bromo-2,2'-[cyclohexane-1,2-diylbis (nitrilomethylidyne)]diphenol, we unexpectedly obtained the title compound (I) whose crystal structure is reported herein.

The molecular structure of (I) is built from two halves related through a two fold axix passing through the middle of the C8-C8^i^ and C10-C10^i^ bonds [(i)= 1-x, 2-y, z)] (Fig. 1). The stereogenic carbon C8 has the R chirality and so the molecule is the enantiomerically pure R,R diastereomer which confirms the synthetic patway used. This molecule is closely related to the (R,R)-N,N'-Bis(5-chlorosalicylidene)- 1,2-cyclohexanediamine compound (Yang *et al.*, 2004).

Intramolecular O-H···N hydrogen bonds also exist in this molecule and thus stabilize the structure (Table 1).

### Experimental

The title compound was synthesized according to the literature (Yang *et al.*, 2004) using the reaction of (R,R)-1,2-cyclohexanediamine, Na_2_SO_4_, and 5-bromon–2-hydroxybenzaldehyde under mild condition. (R,R)-4,4'-Bromo-2,2'-[cyclohexane-1,2-diylbis (nitrilomethylidyne)]diphenol (0.52 g, 1 mmol) was added to a solution of Cd(AC)_2_ .4H_2_O(0.26g, 1mmol) in methanol(20mL). The mixture was heated for 20 hs under reflux with stirring. It was then filtered to give a clear solution, into which diethyl ether vapour was allowed to condense in a closed vessel. After being allowed to stand for a two weeks at room temperature, colorless single crystals were used to measure X-ray diffraction analysis.

### Refinement

The absolute configuration has been deduced from the X-ray structural analyses and confirms the predicted configuration expected from the synthetic pathway.

All H atoms attached to C atoms and O atom were fixed geometrically and treated as riding with C—H = 0.93 Å (aromatic), 0.97 Å (methylene) or 0.98Å (methine) and O—H = 0.82 Å with U_iso_(H) = 1.2U_eq_(C) or U_iso_(H) = 1.5U_eq_(O).

### Crystal data

**Table:**

C_20_H_20_Br_2_N_2_O_2_	*F*(000) = 480
*M**_r_* = 480.20	*D*_x_ = 1.564 Mg m^−^^3^
Orthorhombic, *P*2_1_2_1_2	Mo *K*α radiation, λ = 0.71073 Å
Hall symbol: P 2 2ab	Cell parameters from 1727 reflections
*a* = 5.9323 (16) Å	θ = 2.1–24.9°
*b* = 19.079 (5) Å	µ = 3.99 mm^−^^1^
*c* = 9.009 (2) Å	*T* = 298 K
*V* = 1019.7 (4) Å^3^	Block, colorless
*Z* = 2	0.28 × 0.21 × 0.15 mm

### Data collection

**Table:**

Bruker APEXII area-detector diffractometer	1727 independent reflections
Radiation source: fine-focus sealed tube	1449 reflections with *I* > 2σ(*I*)
graphite	*R*_int_ = 0.024
φ and ω scans	θ_max_ = 24.7°, θ_min_ = 2.1°
Absorption correction: multi-scan (*SADABS*; Bruker, 2005)	*h* = −6→0
*T*_min_ = 0.401, *T*_max_ = 0.586	*k* = −22→22
5912 measured reflections	*l* = −10→0

### Refinement

**Table:**

Refinement on *F*^2^	Secondary atom site location: difference Fourier map
Least-squares matrix: full	Hydrogen site location: inferred from neighbouring sites
*R*[*F*^2^ > 2σ(*F*^2^)] = 0.032	H-atom parameters constrained
*wR*(*F*^2^) = 0.089	*w* = 1/[σ^2^(*F*_o_^2^) + (0.0509*P*)^2^ + 0.1466*P*] where *P* = (*F*_o_^2^ + 2*F*_c_^2^)/3
*S* = 1.06	(Δ/σ)_max_ = 0.001
1727 reflections	Δρ_max_ = 0.34 e Å^−^^3^
119 parameters	Δρ_min_ = −0.39 e Å^−^^3^
0 restraints	Absolute structure: Flack (1983), 681 Friedel pairs
Primary atom site location: structure-invariant direct methods	Flack parameter: 0.018 (18)

### Special details

**Table:**

Geometry. All esds (except the esd in the dihedral angle between two l.s. planes) are estimated using the full covariance matrix. The cell esds are taken into account individually in the estimation of esds in distances, angles and torsion angles; correlations between esds in cell parameters are only used when they are defined by crystal symmetry. An approximate (isotropic) treatment of cell esds is used for estimating esds involving l.s. planes.
Refinement. Refinement of F^2^ against ALL reflections. The weighted R-factor wR and goodness of fit S are based on F^2^, conventional R-factors R are based on F, with F set to zero for negative F^2^. The threshold expression of F^2^ > 2sigma(F^2^) is used only for calculating R-factors(gt) etc. and is not relevant to the choice of reflections for refinement. R-factors based on F^2^ are statistically about twice as large as those based on F, and R- factors based on ALL data will be even larger.

### Fractional atomic coordinates and isotropic or equivalent isotropic displacement parameters (Å^2^)

**Table:**

	*x*	*y*	*z*	*U*_iso_*/*U*_eq_	
Br1	0.48940 (8)	0.71173 (2)	0.82889 (4)	0.0863 (2)	
N1	0.6206 (5)	0.93317 (14)	0.2921 (3)	0.0608 (8)	
O1	0.9786 (5)	0.92461 (14)	0.4597 (4)	0.0835 (8)	
H1	0.8927	0.9446	0.4023	0.125*	
C1	0.6427 (6)	0.77902 (19)	0.7105 (4)	0.0572 (9)	
C2	0.8549 (7)	0.7999 (2)	0.7508 (4)	0.0635 (10)	
H2	0.9242	0.7810	0.8342	0.076*	
C3	0.9636 (6)	0.8492 (2)	0.6663 (4)	0.0677 (10)	
H3	1.1076	0.8637	0.6933	0.081*	
C4	0.8627 (6)	0.87805 (17)	0.5409 (5)	0.0590 (9)	
C5	0.6450 (6)	0.85674 (15)	0.5008 (4)	0.0505 (8)	
C6	0.5368 (6)	0.80627 (17)	0.5877 (3)	0.0533 (8)	
H6	0.3929	0.7911	0.5623	0.064*	
C7	0.5283 (6)	0.88710 (17)	0.3743 (4)	0.0533 (8)	
H7	0.3823	0.8725	0.3529	0.064*	
C8	0.4952 (8)	0.95999 (17)	0.1642 (3)	0.0648 (9)	
H8	0.3376	0.9450	0.1716	0.078*	
C9	0.5975 (9)	0.9300 (2)	0.0232 (5)	0.0878 (15)	
H9A	0.7580	0.9397	0.0222	0.105*	
H9B	0.5779	0.8796	0.0228	0.105*	
C10	0.4915 (13)	0.9604 (2)	−0.1149 (5)	0.1066 (17)	
H10A	0.3342	0.9467	−0.1197	0.128*	
H10B	0.5670	0.9418	−0.2020	0.128*	

### Atomic displacement parameters (Å^2^)

**Table:**

	*U*^11^	*U*^22^	*U*^33^	*U*^12^	*U*^13^	*U*^23^
Br1	0.1059 (4)	0.0925 (3)	0.0605 (3)	−0.0117 (3)	0.0117 (3)	0.01235 (19)
N1	0.0651 (18)	0.0461 (16)	0.071 (2)	0.0081 (14)	−0.0010 (17)	0.0035 (15)
O1	0.0581 (16)	0.0651 (15)	0.127 (2)	−0.0141 (15)	0.001 (2)	0.0131 (15)
C1	0.069 (2)	0.057 (2)	0.0452 (18)	0.0034 (18)	0.0052 (17)	−0.0075 (15)
C2	0.070 (2)	0.069 (2)	0.0509 (19)	0.011 (2)	−0.0112 (19)	−0.0097 (19)
C3	0.051 (2)	0.071 (2)	0.081 (2)	0.0040 (18)	−0.011 (3)	−0.025 (2)
C4	0.054 (2)	0.0445 (18)	0.078 (2)	0.0002 (16)	0.001 (2)	−0.0103 (18)
C5	0.051 (2)	0.0417 (16)	0.058 (2)	0.0042 (14)	0.0024 (18)	−0.0087 (15)
C6	0.050 (2)	0.0580 (18)	0.0516 (17)	−0.0006 (16)	0.0032 (17)	−0.0140 (15)
C7	0.0488 (19)	0.0507 (18)	0.0604 (17)	0.0064 (17)	−0.0017 (18)	−0.0102 (14)
C8	0.074 (2)	0.0500 (17)	0.071 (2)	0.014 (2)	−0.003 (3)	0.0000 (16)
C9	0.126 (4)	0.061 (2)	0.076 (3)	0.030 (3)	−0.004 (3)	−0.007 (2)
C10	0.160 (5)	0.093 (3)	0.067 (2)	0.042 (4)	−0.003 (4)	−0.010 (2)

### Geometric parameters (Å, °)

**Table:**

Br1—C1	1.901 (4)	C5—C7	1.454 (5)
N1—C7	1.273 (5)	C6—H6	0.9300
N1—C8	1.464 (4)	C7—H7	0.9300
O1—C4	1.341 (4)	C8—C9	1.519 (5)
O1—H1	0.8200	C8—C8^i^	1.528 (7)
C1—C2	1.369 (6)	C8—H8	0.9800
C1—C6	1.375 (5)	C9—C10	1.510 (6)
C2—C3	1.371 (5)	C9—H9A	0.9700
C2—H2	0.9300	C9—H9B	0.9700
C3—C4	1.392 (5)	C10—C10^i^	1.515 (10)
C3—H3	0.9300	C10—H10A	0.9700
C4—C5	1.401 (5)	C10—H10B	0.9700
C5—C6	1.397 (5)		
			
C7—N1—C8	118.7 (4)	N1—C7—H7	119.1
C4—O1—H1	109.5	C5—C7—H7	119.1
C2—C1—C6	121.5 (4)	N1—C8—C9	108.9 (3)
C2—C1—Br1	119.2 (3)	N1—C8—C8^i^	109.3 (3)
C6—C1—Br1	119.2 (3)	C9—C8—C8^i^	111.2 (3)
C1—C2—C3	119.0 (4)	N1—C8—H8	109.2
C1—C2—H2	120.5	C9—C8—H8	109.2
C3—C2—H2	120.5	C8^i^—C8—H8	109.2
C2—C3—C4	121.3 (4)	C10—C9—C8	112.2 (4)
C2—C3—H3	119.4	C10—C9—H9A	109.2
C4—C3—H3	119.4	C8—C9—H9A	109.2
O1—C4—C3	119.0 (4)	C10—C9—H9B	109.2
O1—C4—C5	121.6 (4)	C8—C9—H9B	109.2
C3—C4—C5	119.4 (4)	H9A—C9—H9B	107.9
C6—C5—C4	118.6 (3)	C9—C10—C10^i^	110.8 (5)
C6—C5—C7	119.6 (3)	C9—C10—H10A	109.5
C4—C5—C7	121.7 (3)	C10^i^—C10—H10A	109.5
C1—C6—C5	120.1 (3)	C9—C10—H10B	109.5
C1—C6—H6	119.9	C10^i^—C10—H10B	109.5
C5—C6—H6	119.9	H10A—C10—H10B	108.1
N1—C7—C5	121.8 (4)		

Symmetry codes: (i) −*x*+1, −*y*+2, *z*.

### Hydrogen-bond geometry (Å, °)

**Table:**

*D*—H···*A*	*D*—H	H···*A*	*D*···*A*	*D*—H···*A*
O1—H1···N1	0.82	1.91	2.611 (4)	143
